# Premenstrual Dysphoria and Luteal Stress in Dominant-Social-Status Female Macaques

**DOI:** 10.1155/2013/393862

**Published:** 2013-11-28

**Authors:** Mingqi Qiao, Qitao Zhao, Sheng Wei, Huiyun Zhang, Haijun Wang

**Affiliations:** ^1^Institute of Traditional Chinese Medicine Theory, School of Basic Medicine, Shandong University of Traditional Chinese Medicine, Changqing University & Science & Technology Park, Jinan, Shandong 250355, China; ^2^Lab of Traditional Chinese Medicine Classical Theory, Ministry of Education, Shandong University of Traditional Chinese Medicine, Jinan, Shandong 250355, China

## Abstract

The current study aims to extend our previous work to develop nonhuman primate model for prospectively studying the mechanism underlying premenstrual dysphoric disorder (PMDD). Thirty young dominant-status female monkeys were randomly divided into the control group, the model group, and *JQP* group. For two consecutive menstrual cycles, from day 18 to 22, monkeys in the model and *JQP* groups were housed and immobilized singly in specially designed isolation cages for 5-6 hours per day. At the same time, the pharmaceutical interference effect of *jingqianping* (*JQP*) granule, a traditional Chinese medicine specifically used to cure PMDD patients, was tested using monkeys in the *JQP* group. The behavior and facial expressions of monkeys were photographed with an automatic vidicon and were quantitatively analyzed by “*the emotion evaluation scale of female experimental macaque.*” Changes in serum level of progesterone and estradiol were measured with RIA, and serum level of 5-HT, noradrenaline, and dopamine were measured with HPLC. After experiencing mentioned above stress, 70% of monkeys of model group showed PMDD symptoms during three consecutive menstrual cycles. Estradiol and progesterone serum level decreased (*P* < 0.01). Moreover, the peak value of secreted hormones in their follicular phase did not occur. Serum level of 5-HT and dopamine were significantly lower (*P* < 0.01), but the serum noradrenaline level was higher (*P* < 0.01). Moreover, in monkeys administered by *JQP* granule, both PMDD symptoms and the anormal serum level of neurotransmitters could be obviously reversed. This special luteal-phase treatment on dominant-social-status monkeys might be a feasible way to create models mimicking PMDD.

## 1. Introduction

Premenstrual syndrome (PMS) is a poorly understood disorder characterized by moodiness concomitant with psychiatric and physical symptoms during the premenstrual or late-luteal phase of the menstrual cycle. This disorder occurs in up to 20% of reproductive-age women [1]. The main subtype of PMS is premenstrual depression syndrome which primarily shows milder physical symptoms and depressive mood changes [1]. Premenstrual dysphoric disorder (PMDD) can be distinguished from premenstrual depression syndrome and is the most severe subtype of PMS. PMDD affects only 3–9% of women [2–4]. Women with PMDD experience irritability, tension/anxiety, and depression that are so severe that personal and professional roles are frequently disrupted [1, 4–6]. For women with PMDD, the symptoms can be as disabling as a major depressive disorder [1, 4]. Over the years, different hypotheses have been proposed to explain the recurrence of PMDD. Changes in the serum concentrations of ovarian steroids and monoamine neurotransmitters during menstrual cycle are strongly suspected to underlie PMDD, but the exact etiology of this disorder remains unclear [3, 6].

Previously, we established a model of premenstrual depression syndrome in the rhesus monkey (*Macaca mulatta*) by isolating low-social-status young female monkeys with a physical restraint during their luteal phase [7]. These model monkeys presented with premenstrual depressive behavior and expressions that were very similar to the symptoms of women suffering from premenstrual depression syndrome. However, we found that, in high-social-status female monkeys, the same treatment could induce irritable, anxious, and aggressive behavior during their premenstrual phase. Female monkeys, similar to women, show differential sensitivity to stress-induced reproductive dysfunction [8]. It remains unknown whether different endocrine responses can be induced by the same stress in dominant and subordinate young female monkeys.

Social dominance is a fundamental component of both human and nonhuman primate societies. Social status is also a stressor in monkey societies. Socially subordinate females are preferentially susceptible to depressive behavior, and their neurophysiological characteristics may contribute to this susceptibility to depression [9, 10]. Dominant nonhuman primates experience reproductive and health benefits, but the psychiatric and behavioral disorders that they experience remain understudied [11]. It was reported that dominant female cynomolgus monkeys (*M. fascicularis*) exhibit lowercentral serotonergic activity than subordinates [12]. Likewise, dominant female talapoin monkeys have lower concentrations of the serotonin metabolite, 5-hydroxyindole acetic acid (5-HIAA), in their cerebrospinal fluid (CSF) than do low-ranking animals [13]. Gesquiere found that higher-ranking nonhuman primate individuals secrete higher glucocorticoid (stress hormone) levels than lower-ranking ones [11]. A study by Kaplan also showed that dominant monkeys have significantly higher levels of 3-methoxy-4-hydroxyphenylglycol (MHPG, the metabolite of noradrenaline) in their CSF than subordinates [12]. Numerous studies have shown that premenstrual depression patients and animal models have higher serum 5-HT levels [6, 7, 13, 14]; however, PMDD women have lower levels of plasma 5-HT, higher levels of plasma noradrenaline, and higher levels of MHPG in their CSF [15–19]. Thus, we speculated that, owing to their neurophysiological characteristics, dominant monkeys may be more susceptible to irritable behavior, and PMDD models might be developed in these monkeys.

In current study, in order to find out the different endocrine responses induced by the same luteal-phase stress in dominant and subordinate young female monkeys and in order to develop the PMDD monkey models, we isolated and physically restrained dominant young female monkeys during their luteal phase, photographed and quantitatively analyzed the facial expressions, behavior of these monkeys, and examined the secretion pattern of ovarian steroids and monoamine neurotransmitters in the blood between the treated monkeys and control monkeys.

In traditional Chinese medicine, the main subtype of PMS, premenstrual depression syndrome also is termed PMS *liver-qi-yu* syndrome; PMDD, the severe subtype of PMS, is named PMS *liver-qi-ni* syndrome [14]. Furthermore, two famous Chinese ancient recipes, *“xiaoyaoshan” *and* “sinishan,”* had been used to cure PMS *liver-qi-yu* syndrome and *liver-qi-ni* syndrome patients for two thousand years in China. Chinese medicine *Jingqianshu (JQS)* granule and *Jingqianping (JQP)* granule (number of new Chinese patent certificate: Z20040094 and Z20000083) are derived from the above two recipes, respectively [7, 14]. Compound herbal medicine *Jingqianping (JQP)* granule, composed of *Radix paeoniae alba, Rhizoma cyperi, Radix bupleuri, Rhizoma chuanxiong,  Fructus toosendan*, and so on, has emerged as the most effective remedy for PMDD in China for two decades (Xiaoling Sun, et al). In previous studies, we found that most of abnormal changes could be remedied by *jingqianshu* granule in premenstrual depression syndrome model monkeys. In this study, we observed the effect of *jingqianping* granule on our PMDD monkey model.

## 2. Methods

### 2.1. Animals

The protocols of this study were conducted in accordance with standards and guidelines established by the Guide for the Care and Use of Laboratory Animals formulated by the National Institutes of Health and were approved by the Institutional Committee for Animal Care and Use of Shandong University of Traditional Chinese Medicine (approval number: DWSY200710 227).

Ninety healthy young female rhesus monkeys (*M. mulatta*) weighing about 4-5 kg and that were 4–6 years old were purchased from the Suzhou Xishan Zhongke Drugs Research and Development Co., Ltd. Suzhou city, Zhejiang province, China. (SCXK (Su) 2002-0007). These monkeys were captive born and were housed at Jinan zoo (Jinan city, Shandong province, China) in accordance with routine care, management, and assessment protocol. They were bred under controlled conditions: temperature 21-22°C, humidity 55–65%, and 12/12 hour light/dark cycle. The health and general behavior of all monkeys were assessed daily, and each monkey was fed twice daily (at 10 AM and 3 PM, resp.) with a meal of 1-2 steamed breads (250–300 g) of monkey food (composited of corn flour, wheat flour, soy flour, fishmeal, milk powder, sucrose, salt, vegetable tallow, vitamins, and microelement mixture, etc.). The meal was supplemented with both one egg and two pieces of fresh fruit. To provide social enrichment, three animals were housed each cage. Animal maintenance was in accordance with the recommendations of the Guide for the Care and Use of Laboratory Animals and Animal Welfare Act with its subsequent amendments.

All female monkeys in the present study were in their mid reproductive years. According to the volume of sex-organ skin and changes in morphologic characteristics**  **of various vaginal cells in macaques, an entire menstrual cycle (about 28 days) can be divided into four continuous phases: menses (within 2-3 days of the menstrual bleeding), follicular phase (about 10 days from the end of bleeding until ovulation), luteal phase (about 20 days from the end of bleeding), and premenstrual phase (within 5-6 days before menstrual bleeding) [7, 14, 20]. Healthy young female rhesus monkeys bred in Shandong province of China have six menstrual cycles per year (from October of the first year to May of the second year). During the other times of the year, they are in menolipsis phase [21].

### 2.2. Social Status Determination

As described in our previous study, 90 monkeys were randomly assigned to three-member social groups for 2 months, during which their social status stabilized and was determined [7]. The monkey in each group that defeated all other group members was designated the high-social-status monkey. From these 90 monkeys, 30 high-social-status monkeys were chosen for the next phase of the experiment.

### 2.3. Premenstrual Dysphoric Disorder Monkey Model

These 30 monkeys were randomly divided into three groups: the control group, the model group, and *JQP* group (*n* = 10 per  group).   From days 18–22 of their menstrual cycle, monkeys in the model group and *JQP* group were isolated singly (one monkey per cage) and physically restrained in specially designed cages. The moveable doors of these cages were adjusted until the monkeys could not move freely. During the 5 days of this experiment, monkeys were placed in this immovable state for 5-6 hours per day. During other times in the 5 days, monkeys maintained isolated singly in their cages, but they can move freely. The monkeys were treated this way for two consecutive menstrual cycles, during which monkeys in *JQP* group were fed with the Chinese medicine *JQP* granule. In the same time, monkeys in the control group always remained in their three-member social groups.

For the entire experimental period, the behavior and facial expressions of the monkeys were photographed with an automatic vidicon. Moreover, in previous studies, we have established *“the emotion evaluation scale of female experimental macaque”* [22], and the irritable, aggressive facial expression and behavior (including stare, gape, raising eyebrows, roaring, jumping, shaking the cages, attacking other monkeys) were quantitatively analyzed using the scale. For example, the score of attacking other monkeys was permitted to endow any value from 0 to 5 by the trained observer according to his experinece. The emotion of each monkey was evaluated by three observers using the same scale, respectively. Their average value of every entry was analyzed statistically. The final dysphoria factor score was calculated by the formula in *“the emotion evaluation scale of female experimental macaque*,*”* which was an attached file of this article.

There are four kinds of basic emotion in human and higher mammal animals, happiness, depression, anger, and fear. Thus there are four dimensions in *this emotion evaluation scale of female experimental macaque*, happiness dimension, depression dimension, anger dimension, and fear dimension. Anger is the hostility to the persons or things do not meet our expectations, and it is different from anxiety that is the fear of unpredictable things. Irritability is the kind of emotional tendency to anger or depression. According to their behaviors and facial expressions, four kinds of emotion factor score of monkeys all could be calculated by this scale. Dysphoria belongs among the performance of anger [23]. Thus the dysphoria factor score could be regarded as the anger factor score in this emotion evaluation scale.

### 2.4. Hormone Assay

Levels of estradiol and progesterone were detected with a validated radioimmunoassay (RIA) [20]. Measurements were performed according to the manual on RIA kits (Weifang Sanwei Co., Weifang city, Shandong province, china). For two consecutive cycles, blood samples were obtained five times every menstrual cycle (on the 1st day, the 7th day, the 13th day, the 19th day, and the 26th day of every cycle). Blood (2-3 mL) was collected from every monkey each time. Our preliminary experiment showed that this frequency and volume of blood collection have no obvious adverse effects on the monkeys' health. Samples were collected from monkeys by puncturing the hindlimb saphenous vein while they were anesthetized with an im injection of ketamine (ketamine HCl; 15 mg/kg body weight). After centrifugation, the serum was separated and stored at −20°C. All samples were tested at the same time after the confinement treatment had ended. The serum concentration of each sex hormone and monoamine neurotransmitter was expressed as ng/mL or pg/mL. Each value we present is the average of the entire group of 10 monkeys.

### 2.5. Monoamine Neurotransmitter Measurements

Serum levels of monoamine neurotransmitters during the premenstrual phase of the menstrual cycle were determined with high-performance liquid chromatography (HPLC). The experimental procedure and HPLC parameters were as described [24, 25]. Zorbax SB-Cl8 column (4.6 mm × 250 mm, 5 *μ*m, Agilent company) equipped with FLD detection 280 nm and 315 nm, respectively. The mobile phase, comprising of methanol, 0.02 mmoL/L trisodium citrate dihydrate (1 : 99, adjusted to pH 5.0 with hydrochloric acid) was pumped at a rate of 1.0 mL/min. Serum protein was precipitated with a 5% perchloric acid solution and was removed by centrifuging. The recovery rate and RSD of norepinephrine (NE), dopamine (DA), 5-hydroxytryptamine (5-HT) were 97.6%, 98.0%, 96.4%, 103.0%, and 1.1%–1.2%.

### 2.6. Statistical Analysis. 

Data are expressed as the mean ± SE and were analyzed with the Student's *t*-test.

## 3. Results

### 3.1. Behavior Following Stress

**Figure 1:**
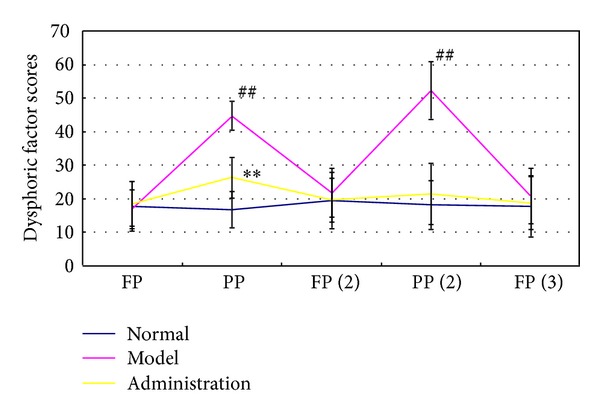
Effect of isolation-induced stress during the luteal phase on the dysphoric factor score of young dominant-social-status female rhesus monkeys (*Macaca mulatta*). The entire menstrual cycle is about 28 days. The first day of menstrual bleeding was defined as the beginning of the menstrual cycle. During days 25–28 is is regarded as the premenstrual phase, and During days 14–19 is regarded as follicular phase, respectively. PP represents the premenstrual phase of the first menstrual cycle since the beginning of the experiment. FP (2) represents the follicular phase of the second menstrual cycle since the beginning of the experiment, and so forth. Values represent mean ± SE (bars) of results for all ten monkeys (^##^the dysphoric factor score in the model group monkeys versus that factor in the control group monkeys *P* < 0.01; **the dysphoric factor score of in the* JQP* group monkeys versus that factor in the model group monkeys).

After this luteal-phase-stress treatment during two consecutive menstrual cycles, 15 of the 20 female monkeys of the model group showed irritable and aggressive behavior and expression during the premenstrual phase or late luteal phase, around days 25–28 in the menstrual cycle. The anger factor score of model monkeys was higher than that score of normal ones (Figure 1, *P* < 0.01) during the premenstrual phase. These symptoms disappeared within 1 or 2 days after the onset of menstrual bleeding. Their anger factor score also returned to normal level (Figure 1, *P* < 0.05). However, these symptoms reoccurred in three subsequent consecutive menstrual cycles after the physical restraint stress had stopped, until these monkeys entered their menolipsis phase. These behavioral characteristics are similar to those of women suffering from PMDD [5–7, 26].

### 3.2. Fluctuating Patterns of Estradiol and Progesterone Levels

**Figure 2:**
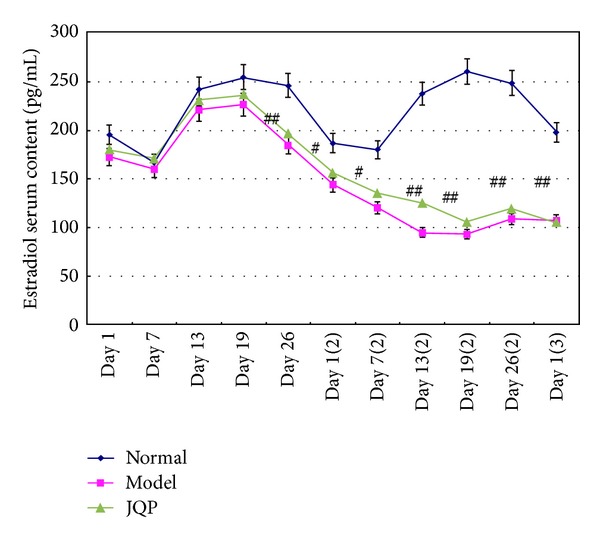
Effect of isolation-induced stress during the luteal phase on the fluctuating patterns of serum estradiol levels in young dominant-social-status female rhesus monkeys (*Macaca mulatta*). Day 1 represents the first day of the first menstrual cycle since the beginning of the experiment. Day 7 (2) represents the seventh day of the second menstrual cycle since the beginning of the experiment, and so forth. The time line of the menstrual cycle and experiment is as described in Figure 2. Values represent the mean ± SE (bars) of results for all ten monkeys (^#^ *P* < 0.05; ^##^ *P* < 0.01). All samples were measured in the same assay.

Our data showed that isolation with physical restraint during the luteal phase obviously affected estradiol secretion in monkeys. In control group monkeys, the serum estradiol level increased to a peak level from days 7 to 19 of the menstrual cycle, the luteal phase. From day 26 of the first menstrual cycle to day 7 of the next menstrual cycle, the serum estradiol level decreased gradually and reached a minimum. Nevertheless, in the model group monkeys, from day 19 (during which they had been isolated and restrained) until day 13 of the next menstrual cycle, the serum estradiol levels markedly decreased and then fluctuated at an obviously lower level. The peak value of estradiol secretion in the luteal phase did not occur (Figure 2).

**Figure 3:**
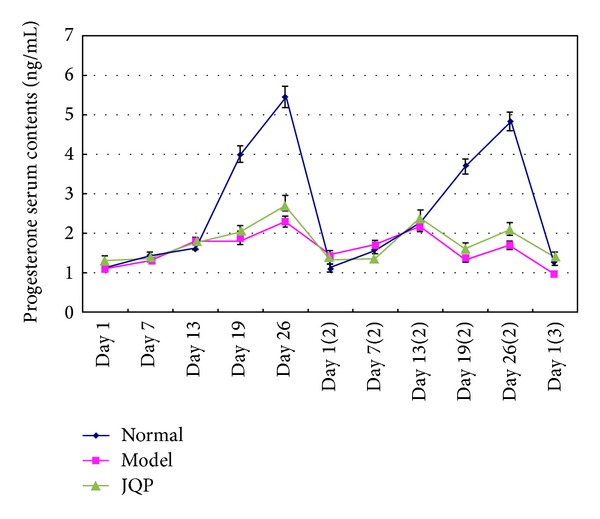
Effect of isolation-induced stress during the luteal phase on the fluctuating patterns of serum progesterone levels in young dominant-social-status female rhesus monkeys (*Macaca mulatta*). Day 1 represents the first day of the first menstrual cycle since the beginning of the experiment. Day 7 (2) represents the seventh day of the second menstrual cycle since the beginning of the experiment, and so forth. The time line of the menstrual cycle and experiment is as described in Figure 2. Values represent the mean ± SE (bars) of results for all ten monkeys (^#^ *P* < 0.05, ^##^ *P* < 0.01). All samples were measured in the same assay.

As shown in Figure 3, compared to the control monkeys, marked changes were observed in the fluctuating pattern of serum progesterone levels in the model group monkeys. During the entire menstrual cycle of control monkeys, serum progesterone levels increased gradually to a peak level on day 26 and then abruptly decreased to a minimal value. The highest and lowest levels of this hormone were reached in the premenstrual and the menses phases, respectively. In model group monkeys, however, serum progesterone levels were lower on day 19 and then increased to a small peak on day 26, which was the premenstrual or late-luteal phase. During the 5 days of isolation and restraint, the serum progesterone levels of the restrained monkeys always fluctuated at a lower level compared with the control monkeys (Figure 3, *P* < 0.05; *P* < 0.01).

### 3.3. Monoamine Neurotransmitter Secretion

**Figure 4:**
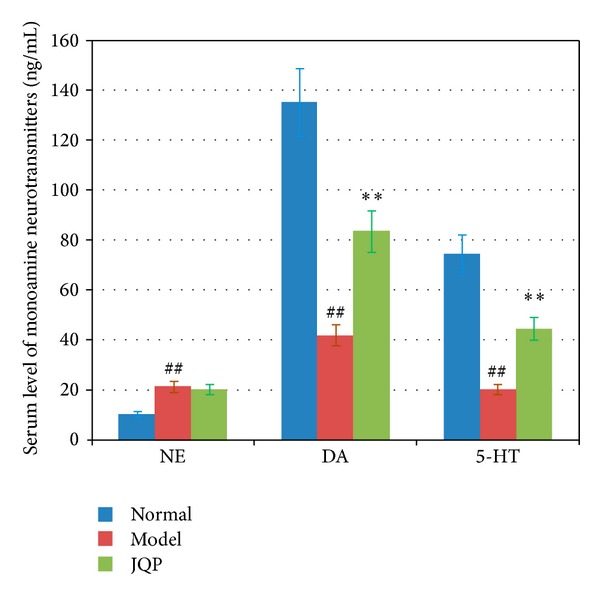
Effect of isolation-induced stress during the luteal phase on serum level of monoamine neurotransmitters in dominant monkeys. NA, 5-HT, and DA. Serum level of noradrenaline, 5-HT, and dopamine. “Normal”, “Model” and “JQP”, indicate the control group, the model group, and the *JQP* group of monkeys, respectively. Values represent means ± SE (bars) of results for all ten monkeys (^##^the serum level of monoamine neurotransmitters in the model group monkeys versus that level in the control group monkeys *P* < 0.01; **the serum level of monoamine neurotransmitters in the* JQP* group monkeys versus that level in the model group monkeys).

Compared with serum levels of control monkeys, the premenstrual serum levels of 5-HT and dopamine in model-group monkeys were significantly lower. However, the serum noradrenaline content was clearly higher (Figure 4, *P* < 0.01).

### 3.4. The Pharmaceutical Interference Effect of Jingqianping Granule

The irritable and aggressive behavior was ameliorated markedly in *JQP* group monkeys. There was no obvious difference observed in the behavior and mood of monkeys between *JQP* group and the normal group, and there is no obvious difference between the anger factor score of monkeys in *JQP* group and that score of monkeys in the control group (Figure 1, *P* < 0.05).

In the serum of monkeys fed with *JQP* granule, level of 5-HT and dopamine were significantly increased (Figure 4, *P* < 0.01). There was no significant difference in the serum estradiol, progesterone, and noradrenaline contents of monkeys between the *JQP* group and the model group. *JQP* granule has no obvious effect on the reproductive function of healthy women [7, 25, 26].

## 4. Discussion 

The most important finding in our current study was that in high-social-status young female monkeys, 5 days of luteal-phase isolation stress induced irritable, anxious, and aggressive behaviors during the premenstrual phase. Moreover, this stress evidently downregulated the secretion of estradiol, progesterone, 5-HT, and dopamine and clearly increased the secretion of noradrenaline. These changes are similar to clinical observations of PMDD patients [5, 7, 26]. These findings support our speculation that dominant monkeys may be preferentially susceptible to anxious behavior and show that a PMDD monkey model can be established by isolating dominant female monkeys during their luteal phase.

Studies have shown that for human and nonhuman female primates, physiological or psychological stresses are associated with alterations in neuroendocrine reactivity to subsequent mental stress and are important precipitating factors of PMDD [2, 5, 27]. Women with PMDD are reported to have greater daily stress [16–18]. Monkeys are highly active animals that live in groups, single caging, and physically, restraint are stressful to them. These forms of stress can be associated with a disruption in their normal menstrual cyclicity [27–29]. In our monkey models, the 5-day luteal-phase stress could account for the neuroendocrine dysregulation and PMDD symptoms. This result provides more evidence in support of the view that stress is strongly associated with PMDD [30, 31].

The cyclical nature of PMDD suggests a hypothesis that dysfunction in hormonal changes related to the menstrual cycle is a primary biological determinant of the disorder. However, the relationship between female sex hormones and PMDD remains unclear. It has been consistently documented that in patients with PMDD, the baseline concentrations of neuroactive steroids are mixed [3, 5, 26]. S. Thys-Jacobs et al. [3] showed that the overall percent of free estradiol across the menstrual cycle and luteal-phase concentrations of free estradiol are significantly lower in PMDD women. N. I. Williams et al. [27] found that in young female rhesus monkeys, short-term stress during their luteal phase results in a significant decrease in progesterone secretion by**  **31%–52%, and cyclic parameters remain abnormal after the stress cycle ends. Our clinical study showed that in Chinese PMDD women, the peak values of secreted estradiol and progesterone in the follicular phase are significantly lower, and there are no secreted peak values in the luteal phase [26]. These studies and our present results demonstrate a strong association between PMDD and consistently low levels of estradiol or progesterone. There is a link between decreased estradiol and progesterone and behavioral indications of our PMDD monkey model; however, the traditional Chinese medicine *JQP* granule specially used to cure PMDD patients has no obvious effect on the serum estradiol and progesterone content of this monkey model, and this medicine might not display its effect by alterating the female hormonal levels of PMDD patients.

This study sheds new light on roles of monoamine transmitters in PMDD. Given the role of 5-HT in mood regulation and in sex steroid-driven behavior modulation, this transmitter promises to be an important factor in PMDD pathophysiology. It was reported that compared to healthy controls, individuals with PMDD show lower serotonergic function during the premenstrual phase [15]. FitzGerald et al. [32] found that in monkeys and apes, there may be a typical luteal-phase increase in serotonin neurotransmission. If this luteal-phase increase is blunted, an increased vulnerability to PMDD may result. Our study also showed that reduced premenstrual serotonergic activity may be a strong trigger of PMDD.

Many studies have shown that PMDD women exhibit greater plasma levels of noradrenaline during their premenstrual phase [17–19, 33]. Consistent with these findings, our monkey data also indicated that diminished hypothalamus-pituitary-adrenal axis function may be a robust characteristic of PMDD. After the luteal isolation stress treatment, the serum level of noradrenaline in monkeys increased notbly, but the effect of *JQP* granule on noradrenaline serum content in the model monkeys is not significant as expected. So this medicine might not affect the mood of monkey model by regulating HPA function.

The dopaminergic system is mainly involved in autonomic manifestations of anxiety [34], but the role of dopamine in PMDD etiology**  **remains unknown. Czoty et al. [35] reported that in the caudate and putamen of female cynomolgus monkeys and women, there are more unoccupied dopamine receptors or decreased dopamine release during the premenstrual phase. Our data showed that, compared with the serum level of dopamine in control group monkeys, this neurotransmitter serum level in model group monkeys decreased observably during premenstrual phase. This evidence indicates that there might be a link between the serum level of dopamine and the mood of monkeys and that dopamine may be involved in regulating premenstrual mood and plays an important role in PMDD occurrence.

One common notion in traditional Chinese medicine is that, there are two main subtypes of PMS, the PMS *liver-qi-yu* syndrome and the PMS *liver-qi-ni* syndrome. These two syndromes of PMS could be cured by different recipes [7, 14]. However, the underlying mechanism remained unclear. In previous studies, we reported that, in premenstrual depression syndrome model monkeys, not only the premenstrual depression symptoms but also the anormal increase of monoamine neurotransmitters serum levels could be remedied by *Jingqianshu (JQS)* granule, the traditional Chinese medicine specially used to cure premenstrual depression patients [14]. In this study, we found that the *JQP* granule specially used to cure PMDD patients in TCM not only could ameliorate the irritable and aggressive behavior but also could significantly increase the serum level of 5-HT and dopamine in PMDD model monkeys. Our studies indicated that the anormal regulation of monoamine neurotransmitters system may play important roles in PMS pathogenesis. Moreover, the mechanism by which Chinese medicine cures this disease may be related with reversing that anormal regulation. *JQS* granule and *JQP* granule might play their roles by inhibiting and activating the monoamine neurotransmitters system, respectively. This granule has no significant effect on the serum content of estradiol and progesterone in our monkey model, and it might not display its effect by alterating the female hormonal levels.

Our previous and present studies showed that it is feasible to create two kinds of monkey models of PMS with the same treatment of dominantand subordinate-social-status monkeys [7, 14]. Such models may not only aid in studies of the mechanisms by which different subtypes of PMS occur but also potentially facilitate development of specific therapies for each subtype of this disease.

## Appendix

### The Emotion Evaluation Scale of Female Experimental Macaque

- Code ______________
- Observation location __________
- Observation time ______________
- Menstrual period ________ ____ d after menstruation
- Observer ___________

(1) *Precautions*

1. Observers should read the option carefully, choose the appropriate answer.
2. Observation time: 9:00 am to 11:00 am.
3. Random observers should carry out their observation at a fixed time, a fixed location.
4. Observers should be away from macaques, so as not to interfere with them.
5. Do not miss any one question.

(2) *Scale Observation Contents*

- Stare score
  (1) None 0
  (2) Have 5
- Eyes' movement slowly score
  (1) None 0
  (2) Common 1.5
  (3) Have 5
- Blink score
  (1) None 0
  (2) Have 5
- Gape score
  (1) None 0
  (2) Have 5
- Bare teeth score
  (1) None 0
  (2) Have 5
- Raising eyebrows and fanning ears score
  (1) None 0
  (2) Have 5
- Hairs score
  (1) Hairs are soft, smooth, tidy and luster 0
  (2) Common 2
  (3) Hairs are chaotic and lack of luster even wool 5
- Roaring score
  (1) None 0
  (2) Have 5
- Irritated easily score
  (1) Irritated hardly 0
  (2) Common 2
  (3) Irritated easily 5
- Jumping score
  (1) None 0
  (2) Have 5
- Shrunk posture score
  (1) Free posture 0
  (2) Common 2
  (3) Shrunk posture 5
- Activity amount score
  (1) Have a small activity amount or substantially not active.  0
  (2) Normal activity amount 3
  (3) Large activity amount, Often in a state of excitement 5
- Bark sadly score
  (1) None 0
  (2) Have 5
- Stay location score
  (1) Stay on the ground mostly.  0
  (2) Stay on a higher location sometimes, on the ground sometimes.  2.5
  (3) Stay on a higher location mostly.  5
- Activity space score
  (1) Activity space is limited, stay on a secluded corner.  0
  (2) Large activity space, can move in their own cages freely.  2
  (3) Not only move in their own cages freely, but also attack macaques in other cages.  5
- Grooming score
  (1) None 0
  (2) Have 5
- Shake their cages score
  (1) None 0
  (2) Have 5
- Attacking other monkeys score
  (1) None 0
  (2) Appearance when external threats occurrence.  3.5
  (3) Appearance even if external threats disappearance.  5
- Tail bending and head hiding score
  (1) None 0
  (2) Have 5
- Stay alone score
  (1) None 0
  (2) Have 5
- Begin to eat after other macaques finished score
  (1) None 0
  (2) Have 5
- Suddenly shriek when they are threatened score
  (1) None 0
  (2) Have 5
- Eat speed score
  (1) Eat calmly 0
  (2) Eat in a hurry 5
- Look directly at opposite side, lips are open and close rapidly score
  (1) None 0
  (2) Have 5
- Urinary and faecal incontinence score
  (1) None 0
  (2) Have 5
- Relaxed and curious, play games regularly score
  (1) None 0
  (2) Have 5
- Snuggle with each other score
  (1) None 0
  (2) Have 5
- Territory behavior score
  (1) None 0
  (2) Have 5

(3) *Calculated Monkey's Emotion Score*

Added the average score of every entry above according to the formulas listed below. Evaluated monkey's emotion according to that score.

Anger  factor  score = A + D + G + H + I + J + L + M + R
Depression  factor  score = B + G + K + M + T + U
Fear  factor  score = C + D + E + K + S + U + V + W + X + Y
Happiness  factor  score = N + O + P + Z + A2 + B2

(4) *Evaluated Monkey's Emotion*

The emotion score reference range of normal female monkey is listed below. If one monkey's emotion score is not within this range, its emotion could be considered as abnormal.

Anger factor score: 21.35 ± 5.13
Depression factor score: 17.96 ± 4.88
Fear factor score: 12.68 ± 2.26
Happiness factor score: 21.25 ± 4.47
